# Bacterial Rotary Export ATPases Are Allosterically Regulated by the Nucleotide Second Messenger Cyclic-di-GMP*[Fn FN2]

**DOI:** 10.1074/jbc.M115.661439

**Published:** 2015-08-11

**Authors:** Eleftheria Trampari, Clare E. M. Stevenson, Richard H. Little, Thomas Wilhelm, David M. Lawson, Jacob G. Malone

**Affiliations:** From the ‡ Molecular Microbiology Department and; the § Biological Chemistry Department, John Innes Centre, Norwich NR4 7UH, United Kingdom,; the ¶ Institute of Food Research, Norwich Research Park, Norwich NR4 7UA, United Kingdom, and; the ‖ School of Biological Sciences, University of East Anglia, Norwich NR4 7TJ, United Kingdom

**Keywords:** ATPase, bacterial signal transduction, cyclic di-GMP (c-di-GMP), Pseudomonas, second messenger, type III secretion system (T3SS), flagellum

## Abstract

The widespread second messenger molecule cyclic di-GMP (cdG) regulates the transition from motile and virulent lifestyles to sessile, biofilm-forming ones in a wide range of bacteria. Many pathogenic and commensal bacterial-host interactions are known to be controlled by cdG signaling. Although the biochemistry of cyclic dinucleotide metabolism is well understood, much remains to be discovered about the downstream signaling pathways that induce bacterial responses upon cdG binding. As part of our ongoing research into the role of cdG signaling in plant-associated *Pseudomonas* species, we carried out an affinity capture screen for cdG binding proteins in the model organism *Pseudomonas fluorescens* SBW25. The flagella export AAA+ ATPase FliI was identified as a result of this screen and subsequently shown to bind specifically to the cdG molecule, with a *K_D_* in the low micromolar range. The interaction between FliI and cdG appears to be very widespread. In addition to FliI homologs from diverse bacterial species, high affinity binding was also observed for the type III secretion system homolog HrcN and the type VI ATPase ClpB2. The addition of cdG was shown to inhibit FliI and HrcN ATPase activity *in vitro*. Finally, a combination of site-specific mutagenesis, mass spectrometry, and *in silico* analysis was used to predict that cdG binds to FliI in a pocket of highly conserved residues at the interface between two FliI subunits. Our results suggest a novel, fundamental role for cdG in controlling the function of multiple important bacterial export pathways, through direct allosteric control of export ATPase proteins.

## Teaser

**Background:** AAA+ ATPase proteins play integral roles in the export apparatus of many bacterial organelles.

**Results:** The second messenger cyclic di-GMP binds specifically to multiple export ATPases at a highly conserved binding site.

**Conclusion:** Cyclic di-GMP binding is central to the function of many different bacterial export complexes.

**Significance:** This profoundly affects our understanding of numerous important bacterial organelles, including flagella, type III, and type VI secretion systems.

## Introduction

*Pseudomonas fluorescens* is a widespread soil bacterium that forms commensal relationships with plant species. Members of the *P. fluorescens* species group nonspecifically colonize the rhizosphere and phyllosphere of many plants and promote plant growth, as well as providing potent antifungal and other biocontrol capabilities (1–3). The related bacterium *Pseudomonas syringae* is a Gram-negative phytopathogen and is responsible for numerous important plant diseases. *P. syringae* produces a large number of species-specific phytotoxins and type III-secreted effector molecules that subvert plant defenses (4, 5) and infects host plants by migration through open stomata and wounds on the plant surface. Two of the most important organelles for efficient host colonization by both commensal and pathogenic *Pseudomonas* sp. are the flagellum and the type III secretion system (T3SS).^3^ Flagella-mediated motility is critical during the initial stages both of infection and benign plant colonization and is required to move through the soil toward plant roots, to colonize plant surfaces, and to migrate into the apoplastic space (6). Type III secretion systems, needle-like structures that inject effector proteins into plant cells, play a critical role in *P. syringae* virulence (4) and have also been shown to be important for rhizosphere colonization by *P. fluorescens* (6).

Assembly of the bacterial flagellum is tightly regulated and proceeds via the export of extracellular subunits through the central pore of the extending complex (7–9). The AAA+ ATPase FliI, together with FliH and FliJ, forms the soluble component of the flagellar export apparatus (8, 10, 11). FliI and FliH form a heterotrimer (FliH_2_-FliI) *in vivo* and along with FliJ deliver export substrates from the cytoplasm to the flagellum export gate. There, FliI forms a hexameric ring and is anchored to the export gate by FliJ and FliH (12). Although the majority of the energy required for flagella formation is provided by the proton motive force, FliI ATPase activity is required for efficient flagella formation and plays a role in the initiation of protein export (13, 14). The secretion apparatus of flagella and T3SS share a conserved core architecture, with many proteins in common including the protein export apparatus (9, 13).

Investigations into the signaling pathways that control interactions between pathogenic and commensal *Pseudomonas* sp. and their host plants have highlighted a central role for the bacterial second messenger cyclic di-GMP (cdG) (15–21). cdG is a ubiquitous regulator of bacterial behavior, controlling the transition between motility and sessility, and chronic and virulent lifestyles in a wide range of bacteria. Recently, cdG has emerged as a crucial factor in the signaling pathways of most bacterial species, determining when, where, and how bacteria form biofilms, progress through the cell cycle, and regulate different aspects of motility and virulence (22). Broadly speaking, cdG production is associated with community behavior phenotypes such as biofilm formation and surface attachment. Conversely, low cdG levels are connected to unicellular, motile, and virulent lifestyles (22). cdG affects cell phenotypes by regulating the expression, production, and activity of different phenotypic output pathways. These outputs are controlled by cdG binding to effectors that function at transcriptional (23), translational (24), and post-translational, allosteric levels (25, 26). Individual phenotypic outputs may be controlled at multiple regulatory stages. For example, the expression of multiple flagella genes are controlled by the cdG-binding transcriptional regulator FleQ (23, 27). Flagella function is also allosterically controlled by cdG, with binding to the basal body-associated proteins YcgR and FlgZ leading to reduced flagella rotation speed in *Escherichia coli* and *Pseudomonas putida*, respectively (28, 29).

cdG-mediated signaling pathways are typically highly complex. Numerous species, including the pseudomonads, contain dozens of metabolic enzymes and display diverse cdG-triggered phenotypes (22). Although the synthesis and degradation of cdG by GGDEF, EAL, and HD-GYP proteins is fairly well understood (30–33), much remains to be discovered about the effector proteins that bind the dinucleotide molecule and elicit downstream responses in the cell. Although several predictable cdG binding folds are known, for example the PilZ (34) and degenerate GGDEF and EAL domains (35–37), cdG is a promiscuous molecule and binds to a diverse range of protein folds. In many cases, these cdG binding motifs are impossible to bioinformatically predict in advance (27, 38–41). This, combined with the complexity of cdG signaling and the diverse array of interconnected, cdG-associated phenotypes (22, 42, 43) suggests that a great many cdG binding proteins still await discovery.

Recent investigations by several research groups have made effective use of biochemical and spectrometric techniques for the isolation and identification of cdG binding proteins (44–46). These studies have both increased our understanding of the phenotypes and cellular functions controlled by this second messenger and substantially expanded the number of recognized binding motifs and protein domains (40, 42, 43). To better understand the role of cdG in the interactions between plant-associated *Pseudomonas* species and their hosts, we used a cdG capture compound assay (45) to screen for cdG binding proteins in the model *P. fluorescens* strain SBW25 (47). Among the proteins we identified in this screen was the flagella export ATPase FliI (PFLU4436). Subsequent biochemical analysis showed that SBW25 FliI specifically binds to cdG with a *K_D_* in the low micromolar range. FliI-cdG binding was not confined to *P. fluorescens* but was also seen for FliI homologs from several other bacterial species, as well as the closely related T3SS export ATPase HrcN from *P. syringae* (PSPTO1400) and the significantly more divergent type VI secretion system secretion ATPase ClpB2 (PFLU6025).

*In vitro* addition of cdG induced a marked, concentration-dependent inhibition of ATPase activity for both FliI and HrcN. However, the association between ATPase activity and cdG binding is not absolute: mutation of critical active site residues in both FliI and HrcN abolishes ATP hydrolysis, whereas dinucleotide binding is unaffected. To further probe the relationship between FliI and cdG, a combination of mass spectrometry and *in silico* analysis was used to predict the FliI dinucleotide binding site. These results suggest that cdG may bind in a pocket of highly conserved residues at the interface between two domains of the FliI hexameric ring. Our results suggest a fundamental new role for the signaling molecule cdG, in the structure and function of multiple widespread and biologically important bacterial export pathways.

## Experimental Procedures

### 

#### 

##### Strains and Growth Conditions

Strains and plasmids are listed in Table 1. Primers are listed in Table 2. Unless otherwise stated, all *P. fluorescens* strains were grown at 28 °C and *E. coli* at 37 °C in lysogenic broth (48), solidified with 1.5% (w/v) agar where appropriate. For protein overexpression, terrific broth was used. Kanamycin was used at 50 μg/ml, carbenicillin was used at 100 μg/ml, and chloramphenicol was used at 30 μg/ml. For inducible plasmids, isopropyl β-d-thiogalactopyranoside was added to a final concentration 0.5 mm as appropriate.

**TABLE 1:** **Strains and plasmids used in this study**

	Description	Reference
**Strains**		
*E. coli* BL21-(DE3)	Sm^R^, K12 *recF143 lacI^q^ lacZ*Δ.*M15*, *xylA*	Novagen
*E. coli* DH5α	*endA*1, *hsdR*17(r_K_-m_K_+), *supE*44, *recA*1, *gyrA* (Nal^R^), *relA*1, Δ(*lacIZYA-argF*) U169, *deoR*, Φ80*dlac*Δ*(lacZ)M15*	Ref. 82
SBW25	Environmental *P. fluorescens* isolate	Ref. 47
*Pto* DC3000	Rif^R^ derivative of *P. syringae* pv. Tomato NCPPB 1106	Ref. 84
*Salmonella enterica* pv. typhimurium	Strain LT2	Ref. 51
*Sinorhizobium meliloti*	Strain 1020	Ref. 83

**Plasmids**		
pET*Nde*M-11	*K_m_*^R^, purification vector, N-terminal His_6_ tag	Ref. 49
pET*Nde*M-11-overexpression vectors	Various overexpression vectors for *fliI* alleles, *hrcN*, and *clpB2* ligated between the NdeI and EcoRI sites of pET*Nde*M-11	This study

**TABLE 2:** **Primers**

Name	Gene Target	Modification	Sequence (5′ → 3′)
A	*PFLU4436* (*fliI*)	None	TTACTTCATATGCGCCTTGATCGCACCAG
B	*PFLU4436* (*fliI*)	None	ATATTCAATTGTTAGCCGCCCGGCGCG
C	*PFLU4436* (*fliI*)	Δ1–18	TTACTTCATATGACATCGTTGCCCGGCCAG
D	*PSPTO1961* (*fliI*)	None	TTACTTCATATGCGCCTTGATCGCGTGAG
E	*PSPTO1961* (*fliI*)	None	AATATCAATTGTCAGCCGCCAGGGG
F	*STY2180* (*fliI*)	None	TTACTTCATATGACCACGCGCCTGACC
G	*STY2180* (*fliI*)	None	AATTGAGAATTCTCACACCGTCGGGAAAAT
H	*SMc03025* (*fliI*)	None	TTACTTCATATGGCACGCGAAGCCGCTG
I	*SMc03026* (*fliI*)	None	AATATCAATTGTCATCCCCTTCCGCGCA
J	*PSPTO1400* (*hrcN*)	None	TTACTTCATATGAACGCCGCACTGAACCAG
K	*PSPTO1400* (*hrcN*)	None	AATATGAATTCTTACTCCGGCAGTTGCGAG
L	*PFLU6025* (*clpB2*)	None	TTACTTCATATGGGTGAAATCAGTCGC
M	*PFLU6025* (*clpB2*)	None	AATATCAATTGTCAATCTGCGTCGC
N	*PFLU4436* (*fliI*)	G176A	CCTGTTCGCCGCTACCGGCGTGG
O	*PFLU4436* (*fliI*)	G176A	CCACGCCGGTAGCGGCGAACAGG
P	*PFLU4436* (*fliI*)	K181A	CGGCGTGGGTGCGTCGGTGTTGC
Q	*PFLU4436* (*fliI*)	K181A	GCAACACCGACGCACCCACGCCG
R	*PFLU4436* (*fliI*)	D265A	GTTGCTGATGGCCTCGCTCACGC
S	*PFLU4436* (*fliI*)	D265A	GCGTGAGCGAGGCCATCAGCAAC
T	*PSPTO1400* (*hrcN*)	K181A	GCGGCAAGGCCACGCTGATG
U	*PSPTO1400* (*hrcN*)	K181A	CATCAGCGTGGCCTTGCCGC

##### Molecular Biology Procedures

Cloning was carried out in accordance with standard molecular biology techniques. The pET*Nde*M-11-*fliI*, *hrcN*, and *clpB2* purification vectors were produced by ligating PCR fragments (amplified with primers A–M from appropriate genomic DNA) between the NdeI and EcoRI sites of plasmid pET*Nde*M-11 (49) as appropriate. Strand overlap extension (50) was used to produce Walker A and Walker B mutants in FliI and HrcN using primers N–U, before cloning into expression vectors as appropriate.

##### cdG Capture Compound Experiments

**FIGURE 1.**
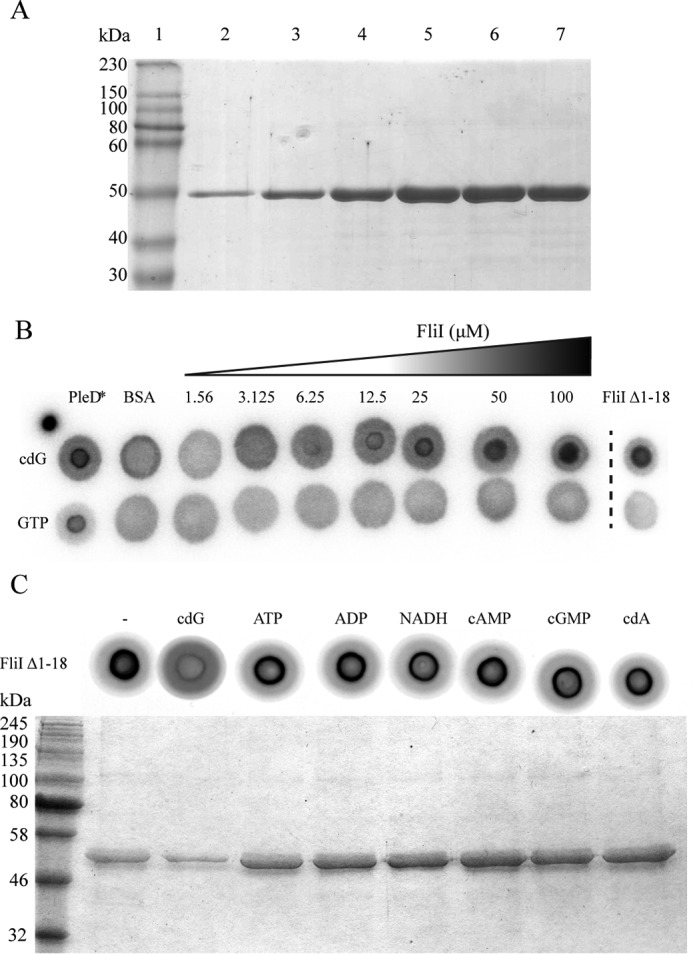
*A*, Coomassie-stained SDS-PAGE gel showing purified FliI_His_ fractions eluted with 500 mm imidazole. *B*, DRaCALA for [^32^P]cdG and [^32^P]GTP binding to increasing concentrations of full-length FliI (FliI_His_). Positive (10 μm PleD*) and negative (10 μm BSA) binding controls are included, as well as N-terminal truncated FliI (10 μm FliI_Δ1–18_). *C*, DRaCALA competition experiment performed on FliI_Δ1–18_. A variety of nucleotides were included in the reaction to test the specificity of cdG binding. SDS-PAGE gel showing protein bound to the capture compound after preincubation with different nucleotides.

Experiments were performed as described by Nesper *et al.* (45). *P. fluorescens* cells were grown in M9 0.4% (w/v) pyruvate medium ± 0.4% (w/v) casamino acids to stationary phase and to mid-logarithmic phase, lysogenic broth to stationary phase, and Kings B medium to logarithmic phase. Cells were collected by centrifugation for 5 min at 5,000 × *g*. The pellet was resuspended in lysis buffer (6.7 mm MES, 6.7 mm HEPES, pH 7.5, 200 mm NaCl, 6.7 mm sodium acetate and 10 mm β-mercaptoethanol) with protease inhibitors and DNase I (Roche). Cells were lysed using a French press (3 × 20,000 p.s.i.), and lysates were centrifuged at 100,000 × *g* for 1 h. The supernatant was then used to identify soluble cdG binding proteins. 600 μg of the soluble protein mixture was used and was mixed with 20 μl of capture buffer (100 mm HEPES, pH 7.5, 250 mm sodium acetate, 50 mm magnesium acetate, 50% (v/v) glycerol), plus 12.5 μl of 10 mm cdG for the control samples. Volumes were adjusted to 100 μl with water, and the reactions were then incubated for 2 h at 4 °C, before UV irradiation for 4 min using a caproBox (Caprotec Bioanalytics, Berlin, Germany). Magnetic streptavidin beads (50 μl) were added with 25 μl of 5× wash buffer (250 mm Tris, pH 7.5, 5 m NaCl, 0.1% (w/v) *n*-octyl-β-glucopyranoside), and the samples were incubated for 45 min at 4 °C on a rotary wheel. The beads were collected with a magnet, and the samples were washed six times with 200 μl of 1× wash buffer. The beads were resuspended in 20 μl of sample buffer, incubated for 10 min at 95 °C, and separated for 10 min on a precast 12% (w/v) SDS acrylamide gel at 100 V. Protein bands were then excised and sent for mass spectrometric analysis. The same protocol was followed for the competition experiment (see Fig. 1*C*). Similarly to the controls, 1 mm of each nucleotide was added to a protein mixture of 10 μm and preincubated for 1 h before the addition of the capture compound (10 μm).

##### Protein Purification

*E. coli* BL21-(DE3) pLysS overexpression cultures were inoculated from overnight cultures in a 1:100 ratio and grown at 37 °C to an *OD*_600_ of 0.4, before protein expression was induced overnight with 0.5 mm isopropyl β-d-thiogalactopyranoside at 18 °C. Cells were then lysed by French press (3 × 20,000 p.s.i.) and centrifuged, and the proteins purified were from the supernatant by nickel-nitrilotriacetic acid chromatography. 1-ml HiTrap chelating HP columns (Amersham Biosciences) were equilibrated with 10 volumes of washing buffer (20 mm HEPES, pH 7.5, 250 mm NaCl, 2 mm MgCl_2_, and 2.5% (v/v) glycerol, pH 7.5) and loaded with cell lysate. Following protein immobilization, the column was washed with 10 volumes of buffer containing 50 mm imidazole, before proteins were eluted using 500 mm imidazole buffer in a single step elution.

##### Differential Radial Capillary of Ligand Assay (DRaCALA)

The method was performed as described by Roelofs *et al.* (44). Purified PleD* (52) was used to synthesize radiolabeled cdG from [γ-^32^P]GTP. The assays were conducted using increasing concentrations of purified FliI proteins mixed with 4 nm radiolabeled cdG in each case. Samples were incubated for 2 min at room temperature with [γ-^32^P]cdG in reaction buffer (25 mm Tris, 250 mm NaCl, 10 mm MgCl_2_, 5 mm β-mercaptoethanol, pH 7.5). 5 μl of each sample were then spotted on nitrocellulose, samples were dried, and results were visualized using a PhosphorImager screen. In some cases 2′-Fluo-AHC-c-diGMP was used (BioLog 009) as an alternative to [γ-^32^P]cdG, at a concentration of 0.6 μm. The results were visualized using a charge-coupled device camera. For the competition experiments, 1 mm of each nucleotide was mixed with 10 μm of FliI_Δ1–18_ and incubated for 30 min before the addition of the fluorescent cdG.

##### Surface Plasmon Resonance (SPR)

SPR experiments were conducted at 25 °C with a Biacore T200 system (GE Healthcare) using a streptavidin SA sensor chip (GE Healthcare), which has four flow cells each containing SA preimmobilized to a carboxymethylated dextran matrix. Flow cells 1 and 3 were kept blank to use for reference subtraction. The chip was first washed three times with 1 m NaCl, 50 mm NaOH to remove any unconjugated streptavidin. 100 nm biotinylated cdG (BioLog B098) was immobilized on flow cells 2 and 4 of the streptavidin chip at a 50-response unit immobilization level with a flow rate of 5 μl/min. Soluble proteins at the required concentrations were prepared in SPR buffer (10 mm HEPES, 150 mm NaCl, 0.1% (v/v) Tween 20, 2 mm MgCl_2_) by adjusting the pH for the different proteins. For FliI_His_, FliI_His_ mutants, HrcN_His_, HrcN_His_ mutants, and ClpB2, the optimal pH was 6.5, whereas for FliI_SeT_, the optimal pH was 7.5, and for FliI_Δ1–18_ it was 5.5. Samples were injected with a flow rate of 5 μl/min over the reference and cdG cells for either 60, 90, or 120 s depending on their saturation level followed by buffer flow for either 60 or 90 s. The chip was washed at the end of each cycle with 1 m NaCl. Replicates for each protein concentration were included as appropriate. In certain cases (*e.g.* FliI_SeT_), protein precipitation at higher concentrations prevented the acquisition of a saturated binding curve. In these cases, a representative data set is presented from at least three independent repetitions. All sensorgrams were analyzed using Biacore T200 BiaEvaluation software version 1.0 (GE Healthcare). The data were then plotted using Microsoft Excel and GraphPad Prism.

##### Linked Pyruvate Kinase/Lactate Dehydrogenase ATPase Activity Assay

ATPase activity was measured indirectly by monitoring NADH oxidation. The reaction buffer consisted of 50 mm Tris-Cl, pH 8.0, 2 mm MgCl_2_, 1 mm DTT, and 10 mm KCl. Each reaction contained 5 mm NADH in 10 mm NaOH, 80 mm phosphoenolpyruvic acid, 1.5 μl of pyruvate kinase/lactate dehydrogenase (Sigma), and appropriate concentrations of FliI/HrcN and cdG and was initiated by the addition of ATP. Enzyme kinetics were determined by measuring *A*_340_ at 1-min intervals. Kinetic parameters were calculated by plotting the specific activity of the enzyme (nmol of ATP hydrolyzed/min/mg of protein) *versus* ATP concentration and by fitting the nonlinear enzyme kinetics model (Hill equation) in GraphPad Prism.

##### Mass Spectrometry of Cross-linked FliI

10 μm FliI_His_ protein was incubated with 10 μm cdG capture compound (Caprotec) and cross-linked in a UV Stratalinker on ice for 4 min. Cross-linked sample was then separated from non-cross-linked using magnetic beads as described for the capture compound screen (above). Cross-linked FliI-cdG and a non-cross-linked control sample were then run into an SDS gel, and FliI bands were excised for protein identification. Samples were analyzed by Nano-LC-MS/MS on an Orbitrap Fusion^TM^ Tribrid^TM^ mass spectrometer coupled to an UltiMate® 3000 RSLCnano LC system (Thermo Scientific, Hemel Hempstead, UK). The sample was separated on a PepMap^TM^ 100 C18 LC Column (C18, 2 μm, 500 × 0.75 mm; Thermo) using a gradient of 0.75% (v/v) min^−1^ acetonitrile from 6% to 40% (v/v) in water, 0.1% (v/v) formic acid at a flow rate of 0.3 μl min^−1^ and infused directly into the mass spectrometer. The mass spectrometer was run in positive ion mode, with no quad isolation, at 120K resolution over the mass range 350–1800 (*m*/*z*) for the precursor scans (Orbitrap). One microscan of 50 ms with an AGC target of 2e^5^ was used. MS2 threshold was set to 1.5e^4^, and precursors were fragmented by both CID and HCD with CE = 30 and an isolation window of 1.6 Da (quadrupole) using the automatic maximum speed option with ion injection for all available parallelizable time. Dynamic exclusion was set to 1 count and 30 s. Recalibrated peaklists were generated using MaxQuant 1.5.2.8, and the database search was performed with the merged HCD and CID peaklists using Mascot 2.4 (Matrixscience, London, UK). The search was performed with a precursor tolerance of 6 ppm and a fragment tolerance of 0.6 Da on a partial *E. coli* database, to which the expected FliI_His_ protein sequence was added. The enzyme was set to trypsin/P with a maximum of two allowed missed cleavages. Carbamidomethyl (C) was set as fixed modification, and oxidation (M) and acetylation (Protein N-term) were used as variable modifications. The Mascot search results were imported into Scaffold 4.4.1.1 using identification probabilities of 99 and 95% for proteins and peptides.

##### Homology Model Production

A homology model of a *P. fluorescens* FliI monomer was created by the Phyre2 server (53) using the crystal structure of FliI from *Salmonella enterica* as a template (Protein Data Bank accession code 2DPY; 63% amino acid sequence identity) (54). FliI is predicted to be structurally homologous to the α and β subunits of F_1_-ATPase, and the latter forms a hexameric ring of alternating α and β subunits around a single copy of a γ subunit. Using the secondary structure matching algorithm (55) within the program COOT (56), a model of a FliI hexamer was generated by superposing six copies of the monomer onto each of the α and β subunits of the bovine F_1_-ATPase α_3_β_3_γ complex (Protein Data Bank accession code 2JIZ) (57). All structural figures were prepared using CCP4 mg (58).

##### Mass Spectrometry-Peak Shift Analysis (MS-PSA)

MS-PSA analysis of the sample containing the treated protein FliI_His_ (*i.e.* cross-linked to cdG) was performed as described previously (44). We further improved the method to specifically search for spectra relations (*i.e.* modified *versus* unmodified peptide) between spectra from two different samples. Accordingly, we found many related peptides between the pure (untreated) FliI_His_ sample and the treated sample (FliI_His_ cross-linked to cdG), particularly for the peptide NVLLLMDSLTR. The following MS-PSA parameters were used for both analyses: num = 8 pmr = 0.001 t1 = 5 t2 = 100 fmr = 0.5 mnds = 2 clusterq = True signif = 1 tol = 1 outl = 0.1 nmfp = 20 mofp = 9 mnspg = 2 csf = 2 peakfreq = 2 nummods = 1 pwss = 4 cp = 3 qual1 = 0.2 qual2 = 0.2 qual1pl2 = 0.5 mrms = 150 maxdev = 1. MS-PSA parameters are defined in Fig. S1 of Ref. 44.

## Results

### 

#### 

##### The Flagellar ATPase FliI Binds Specifically to cdG

**FIGURE 2.**
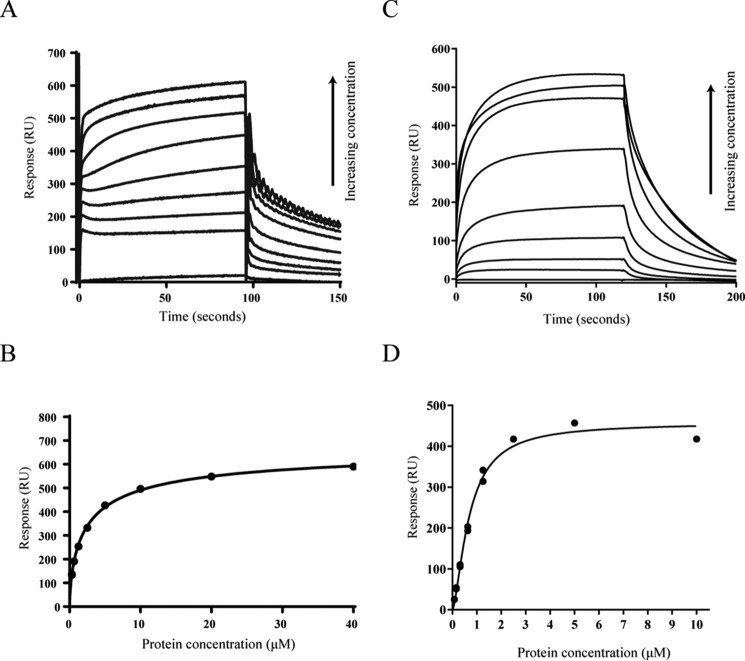
*A*, SPR sensorgrams showing affinity measurements for FliI_His_ binding to biotinylated cdG. A range of FliI_His_ concentrations was used (0.312, 0.625, 1.25, 2.5, 5, 10, 20, and 40 μm), and concentration replicates were included as appropriate together with buffer only and BSA controls. The protein binding and dissociation phases for all sensorgrams are shown. *B*, affinity fit for FliI_His_-cdG binding. The binding response for each concentration was recorded 4 s before the end of the injection, and the *K_D_* values for FliI_His_ binding to cdG (2.4 ± 0.2 μm) were calculated using the BiaEvaluation software and confirmed by GraphPad Prism. *C*, SPR sensorgrams showing affinity measurements for FliI_Δ1–18_ binding to biotinylated cdG. A range of protein concentrations was used (0.078, 0.156, 0.3125, 0.625, 1.25, 2.5, 5, and 10 μm), and concentration replicates were included as appropriate together with buffer only and BSA controls. The protein binding and dissociation phases for all sensorgrams are shown. *D*, affinity fit for FliI_Δ1–18_-cdG binding. Binding responses were measured 4 s before the end of the injection, and the *K_D_* values for FliI_Δ1–18_ binding to cdG (0.8 ± 0.03 μm) were calculated using the BiaEvaluation software and confirmed by GraphPad Prism.

As part of our ongoing efforts to define the cdG regulon of *P. fluorescens* SBW25, we carried out a series of screening experiments for cdG binding proteins using a cdG capture compound assay (45) (Caprotec). These experiments identified homologs of confirmed binding proteins, including FleQ (23) and WspR (59), as well as several uncharacterized PilZ, GGDEF, and EAL domains and numerous proteins for which no previous experimental or predicted link to cdG signaling had been made. Among these previously unidentified cdG targets, the flagellar export protein FliI was identified in screens conducted under a number of different experimental conditions (Kings B medium log phase, lysogenic broth stationary phase, and M9 pyruvate + casamino acids). In addition to the suppression of flagellar gene expression (23) and flagellum rotation (29, 60), FliI-cdG binding suggests a central, previously unsuspected role for cdG in the regulation of flagellum protein export and assembly. Consequently, we selected FliI for further biochemical analysis. First, to confirm that SBW25 FliI binds to cdG *in vitro*, the full-length, His-tagged protein (FliI_His_) was purified (Fig. 1*A*), and nucleotide binding was tested using the DRaCALA binding assay (44). FliI_His_ bound strongly to ^32^PcdG but did not bind to ^32^PGTP even at far higher protein concentrations (Fig. 1*B*). To further test the specificity of FliI-cdG binding, competitive DRaCALA and capture compound experiments were performed (Fig. 1*C*). A variety of nucleotides (cyclic di-GMP, ADP, ATP, NADH, cAMP, cGMP, and cyclic di-AMP) were added in excess to compete the 2′-Fluo-AHC-c-diGMP and capture compound, respectively. In both experiments, binding was abolished only with the addition of cdG, strongly suggesting that cdG binding by FliI is specific. Our initial attempts to define the biochemical parameters of cdG binding to FliI_His_ used isothermal titration calorimetry. This technique showed tight, concentration-dependent cdG binding with a *K_D_* of ∼10 μm (data not shown). However, we were unable to refine the isothermal titration calorimetry protocol sufficiently to produce publishable data. Consequently, we turned to SPR to examine FliI_His_ binding to biotinylated cdG. In this experiment, FliI_His_ bound to the cdG chip in a concentration-dependent manner with a *K_D_* of 2.4 ± 0.2 μm (Fig. 2, *A* and *B*, and Table 3).

**TABLE 3:** **Binding affinity data for tested FliI, HrcN, and ClpB2 variants** Dissociation constant (*K_D_*) values for the ATPase proteins analyzed in this study are shown. Binding to FliI_SeT_ was concentration-dependent but did not saturate in the kinetic experiment.

	*K_D_*
	μ*m*
FliI_His_ (full-length)	2.4 ± 0.2
FliI_Δ1–18_ (N-terminally truncated)	0.8 ± 0.03
FliI_Δ1–18_ R170H	Not determined
FliI_Δ1–18_ E208Q	Not determined
FliI_Δ1–18_ R337H	Not determined
FliI_Pto_ (*Pto* DC3000)	7.6 ± 0.8
FliI_SeT_ (*S. enterica*)	Not determined
FliI_Sm_ (*S. meliloti*)	3.2 ± 0.7
HrcN_His_	3.2 ± 0.2
ClpB2_His_	9.5 ± 0.5
FliI G176A (Walker A mutant)	11.0 ± 1.1
FliI K181A (Walker A mutant)	2.2 ± 0.2
HrcN K181A (Walker A mutant)	3.8 ± 0.4
FliI D265A (Walker B mutant)	4.5 ± 0.2

Much of the structural and functional analysis of FliI to date has been conducted in *S. enterica*, whose purified FliI homolog is unstable *in vitro* unless the N terminus of the protein is truncated (61). This modification affects FliI multimerization, with the truncated allele unable to form hexameric complexes *in vitro* (62). To examine the effects of removing the FliI N terminus on protein-ligand interactions, a truncated FliI allele missing the first 18 residues (FliI_Δ1–18_) was purified and analyzed alongside full-length FliI_His_. The FliI_Δ1–18_ allele bound tightly and specifically to cdG in both DRaCALA and SPR experiments, with a dissociation constant of 0.75 ± 0.03 μm (Figs. 1*B* and 2, *C* and *D*). The binding constants for both full-length and truncated FliI fall comfortably within the affinity range of previously characterized cdG binding proteins (between the low nanomolar range and 10–15 μm (63)), indicating that FliI-cdG binding is likely to occur in *P. fluorescens* under physiologically relevant conditions.

##### FliI Homologs from Diverse Bacterial Species and the Export ATPases of Type III and Type VI Secretion Systems Bind cdG at Physiological Concentrations

**FIGURE 3.**
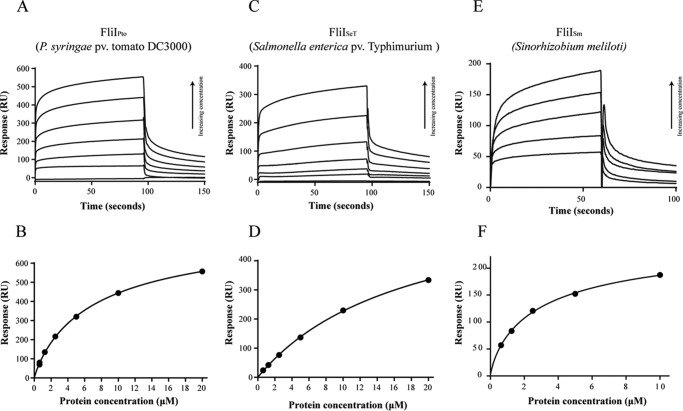
*A* and *B*, SPR sensorgrams and resulting affinity fit for FliI_Pto_ binding to biotinylated cdG. *C* and *D*, SPR sensorgrams and affinity fit for FliI_SeT_ binding to biotinylated cdG. *E* and *F*, SPR sensorgrams and affinity fit for FliI_Sm_ binding to biotinylated cdG. In all three cases, a range of protein concentrations was used (0.625, 1.25, 2.5, 5, 10, and for FliI_Pto_/FliI_SeT_ 20 μm), and concentration replicates were included as appropriate together with buffer only and BSA controls. The protein binding and dissociation phases for all sensorgrams are shown. For the affinity fits, binding responses were measured 4 s before the end of the injection, and *K_D_* values for each protein were calculated using the BiaEvaluation software and confirmed by GraphPad Prism (Table 3).

Flagella-driven motility, and hence FliI-mediated export, is ubiquitous among Gram-negative bacteria. To investigate whether cdG-binding to FliI is similarly widespread, full-length FliI homologs from several bacterial species were cloned, expressed, purified, and then tested for cdG binding using SPR. FliI homologs were selected from human and plant pathogens, as well as commensal and symbiotic plant growth-promoting organisms. The tested FliI homologs included representatives from the α- and γ-proteobacterial classes and both monotrichous and polyflagellated bacteria. Concentration-dependent cdG binding was detected for full-length FliI alleles from the phytopathogen *P. syringae* pv. *tomato* (*Pto*) DC3000 (FliI_Pto_), the human pathogen *S. enterica* serovar typhimurium (FliI_SeT_), and the nitrogen-fixing symbiont *Sinorhizobium meliloti* (FliI_Sm_) (Fig. 3 and Table 3). Despite a reasonably high degree of *fliI* amino acid sequence divergence (SBW25 and *S. meliloti fliI* share only 35.4% identity) and significant differences in flagella regulation and cdG signaling between the tested species, all four FliI homologs bound to the dinucleotide molecule with affinities well within the expected physiological range of intracellular cdG concentrations.

**FIGURE 4.**
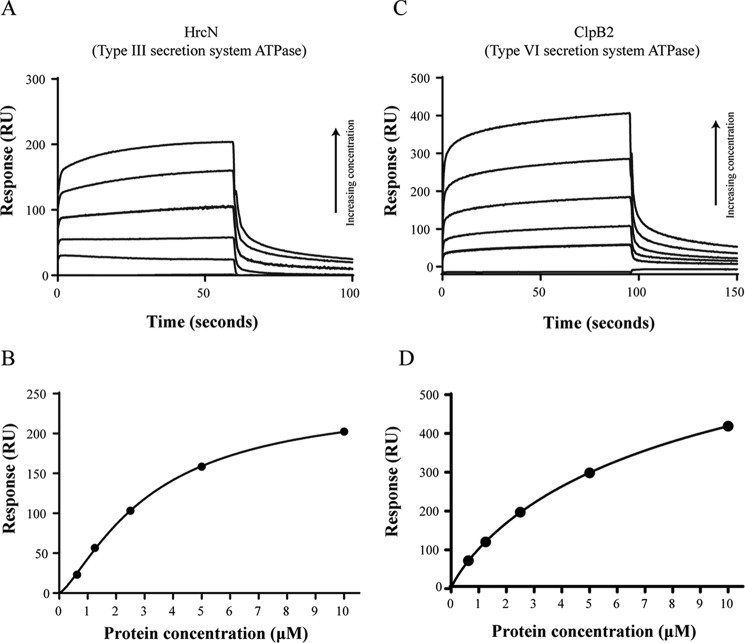
*A* and *B*, SPR sensorgram and resulting affinity fit for HrcN (type III export ATPase) binding to biotinylated cdG. *C* and *D*, SPR sensorgram and resulting affinity fit for ClpB2 (Type VI export ATPase) binding to biotinylated cdG. In both cases, a range of protein concentrations was used (0.625, 1.25, 2.5, 5, and 10 μm), and concentration replicates were included as appropriate together with buffer only and BSA controls. The protein binding and dissociation phases for all sensorgrams are shown. For the affinity fits, binding responses were measured 4 s before the end of the injection, and *K_D_* values for each protein were calculated using the BiaEvaluation software and confirmed by GraphPad (Table 3).

The export apparatus of the bacterial flagellum is closely related to that of the T3SS, with both complexes sharing a common ancestor (8). Furthermore, cdG has been associated with the control of T3SS function in the opportunistic pathogen *Pseudomonas aeruginosa* (64, 65), although the mechanism of this regulation is currently unclear. In light of this, our data for FliI-cdG binding implicate the T3SS export ATPase HrcN as a further potential cdG-binding target. To test this, we purified the full-length, His-tagged protein from *Pto* DC3000 (HrcN_His_) and examined cdG binding using SPR. As predicted, HrcN_His_ also bound strongly to cdG, with a dissociation constant of 3.2 ± 0.2 μm (Fig. 4, *A* and *B*). The type VI secretion system export ATPase (ClpB2) is far more distantly related to FliI, in terms of both primary sequence and organization of the ATPase subunits within the type VI secretion complex (66). Nonetheless, as ClpB2 is a rotary ATPase and type VI secretion is known to be under reciprocal, cdG-linked control with type III secretion (65), full-length ClpB2 (ClpB2_His_) was purified and tested for cdG binding. To our surprise, ClpB2 also displayed strong, concentration-dependent binding to the cdG with a physiologically relevant binding affinity of 9.5 ± 0.5 μm (Fig. 4, *C* and *D*). These data strongly suggest that binding to the cdG second messenger is a widespread characteristic across diverse rotary ATPase export proteins.

##### Addition of cdG Inhibits FliI and HrcN ATPase Activity

**FIGURE 5.**
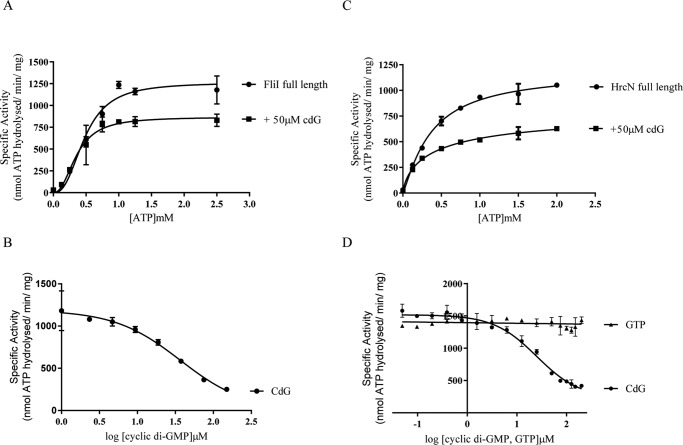
*A*, ATPase activity for FliI_His_ ± 50 μm cdG. FliI_His_ specific activity (nmol ATP hydrolyzed/min/mg protein) is shown for increasing ATP concentrations. Addition of cdG causes a decrease of the *V*_max_ of FliI_His_ ATPase activity. *B*, IC_50_ curve for FliI_His_ ATPase activity upon addition of increasing cdG concentrations. A constant concentration of ATP (1 mm) was included alongside 1 μg of FliI_His_ protein. *C* and *D*, ATPase activity ± 50 μm cdG, and IC_50_ curve upon addition of increasing cdG concentrations, for HrcN. All parameters remain the same as in *A*. The IC_50_ curve also includes results for GTP titration showing no ATPase inhibition.

The established model for cdG function associates increased dinucleotide levels with reduced motility and virulence (22). This has been shown to be the case for both *P. fluorescens* (67) and *P. syringae* (17, 18, 21). Consequently, we hypothesized that cdG binding may negatively affect the ATP-dependent export activity of FliI and/or HrcN. To examine the effect of cdG binding on FliI/HrcN ATPase activity, pyruvate kinase/lactate dehydrogenase-linked ATPase activity assays were conducted for the full-length protein alleles FliI_His_ and HrcN_His_. Purified FliI_His_ metabolized ATP with a *K_m_* of 0.48 ± 0.03 μm and a *V*_max_ of 1262 ± 54.46 nm ATP/min/mg. Addition of 50 μm cdG led to a noticeable drop in *V*_max_, to 867.2 ± 51.65 nm ATP/min/mg (Fig. 5*A*). The IC_50_ of cdG for FliI_His_ (36.7 ± 1.13 μm) was then determined by increasing cdG levels while maintaining a constant ATP concentration in the reaction (Fig. 5*B*). Similar cdG inhibitory activity was seen for FliI_Δ1–18_, which metabolized ATP with *K_m_* and *V*_max_ values of 0.45 ± 0.04 μm and 691.5 ± 41.90 nm ATP/min/mg without cdG. Upon addition of 50 μm of the dinucleotide molecule, *V*_max_ dropped to 375.4 ± 35.34 nm ATP/min/mg. FliI_Δ1–18_ has an IC_50_ value of 48.8 ± 0.159 μm. In agreement with DRaCALA results seen for FliI_His_ (Fig. 1*B*), addition of GTP produced no change in ATPase activity, supporting a specific inhibitory role for cdG toward FliI ATPase activity (data not shown). Full-length HrcN showed a similar degree of ATPase inhibition to FliI, with *V*_max_ dropping from 1183 ± 68.2 to 832.4 ± 119.7 nm ATP/min/mg upon addition of 50 μm cdG, with an IC_50_ of 25.11 ± 1.14 μm (Fig. 5, *C* and *D*). Addition of GTP had no effect on HrcN_His_ ATPase activity (Fig. 5*D*), arguing once again for specific cdG inhibition of HrcN ATPase activity.

##### ATPase Activity and cdG Binding Are Uncoupled by Mutations in the FliI/HrcN Walker A and B Motifs

**FIGURE 6.**
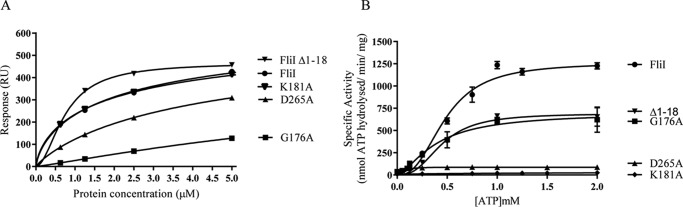
*A*, affinity fit for cdG binding to different FliI alleles (FliI_His_, Δ1–18, K181A, D265A, and G176A). Sensorgrams obtained using biotinylated cdG (Figs. 1*D* and 2*B*) were used to calculate the *K_D_* values for FliI binding to cdG (Table 3). At each protein concentration, the responses were recorded 4 s before the end of the injection. *B*, ATPase activity for different FliI alleles. Protein specific activity in each case (nmol ATP hydrolyzed/min/mg protein) is shown for increasing ATP concentrations.

To further examine the relationship between ATP hydrolysis and cdG binding for FliI and HrcN, a series of full-length alleles were produced with residue substitutions in the conserved Walker A and Walker B motifs (68, 69) of the ATPase active site. The variant proteins were then purified and subjected to SPR analysis and ATPase activity assays as described above. Substitution of the critical FliI Walker A lysine residue for alanine (K181A) had very little effect on cdG binding affinity (Fig. 6*A* and Table 3) but entirely abolished ATPase activity (Fig. 6*B*). Conversely, substitution of the first conserved Walker A glycine to alanine (G176A) led to a mild reduction in FliI ATPase activity but almost entirely abolished the ability of the protein to bind cdG. Finally, a Walker B motif (D265A) substitution in FliI resulted in the complete abolition of ATPase activity but only a relatively small drop in the cdG binding affinity of the protein (Fig. 6, *A* and *B*, and Table 3). This is consistent with our previous results, which show that neither ATP nor ADP have any effect on cdG binding when included in DRaCALA assays (Fig. 1*C*). These results indicate that cdG binding and ATPase activity may be uncoupled.

##### Identifying the Site of cdG Capture Compound Binding in FliI

**FIGURE 7.**
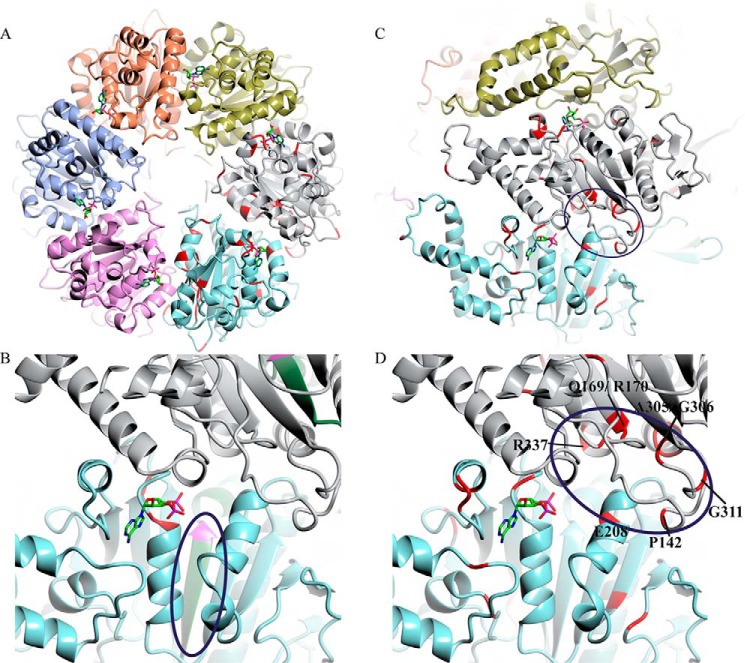
*A*, homology model of the predicted hexameric form of SBW25 FliI, based on the crystal structure of FliI from *S. typhimurium* (Protein Data Bank code 2DPY). Conserved residues between the six cdG-binding proteins tested in this study are marked in *red* (on the *gray* and *cyan*-colored subunits only), and ADP (stick model; taken from template structure) is shown bound at the interfaces between the individual FliI subunits. *B*, close-up of the interface between two FliI subunits, showing the NVLLLMDSLTR peptide implicated in cdG capture compound binding (*circled*, in *green*) and the conserved Walker B aspartate (Asp-265) in *pink. C*, locations of conserved residues between the six cdG-binding proteins tested in this study (*red*). *D*, close-up of the proposed cdG binding pocket (*circled*). Conserved residues suggested to form the cdG binding site are labeled.

To further investigate the site of cdG binding on FliI, we constructed a homology model for SBW25 FliI based on the crystal structure of its *Salmonella* homolog (54) (Fig. 7). The location of ATP and the conserved residues across the six cdG-binding ATPases in this study were then mapped onto the model, and a predicted FliI hexameric complex was produced (Fig. 7*A*). Next, purified FliI_His_ was incubated and UV cross-linked to the cdG capture compound. Following tryptic digestion and mass spectrometry, mass-shifted peptides were identified using MS-PSA, a recently developed analysis method for the identification of unexpected/unknown peptide modifications (70) (supplemental Fig. S1). Two MS-PSA analyses were performed, treated (*i.e.* cross-linked to cdG) *versus* untreated FliI_His_ (S1A) and treated FliI_His_ alone (S1B). In the treated sample, we expected to identify both pure FliI_His_ and FliI_His_ with bound cdG. Accordingly, many spectra relations corresponding to modified and unmodified peptides were identified (supplemental Fig. S1). Importantly, the most densely modified peptide following cdG capture compound cross-linking comprised residues 259–269 (NVLLLMDSLTR; supplemental Fig. S1). We identified 36 spectra relations where the lighter peptide was Mascot annotated NVLLLMDSLTR, and the heavier not-annotated partner carried a modification of >150 Da. By only comparing spectra between the treated and untreated sample, we identified 52 corresponding NVLLLMDSLTR spectra relations. The NVLLLMDSLTR peptide represents the central strand of a β-sheet at the core of the SBW25 FliI homology model, plus short loops at either end (Fig. 7*B*, *green*). The C terminus of the β-strand also contains the conserved aspartate (Asp-265) of the Walker B motif (Fig. 7*B*, *pink*). Interestingly, the end of the capture compound cross-linked peptide emerges close to a cluster of highly conserved residues that could form a pocket at the interface between two FliI subunits in our model (Fig. 7, *C* and *D*, *red*). As well as several glycine and proline residues, this conserved pocket contains two arginines (Arg-170 and Arg-337) from one subunit and a glutamate (Glu-208) from the second. Both arginine and glutamate are highly important for dinucleotide binding in all previously characterized cdG binding proteins (40, 42).

**FIGURE 8.**
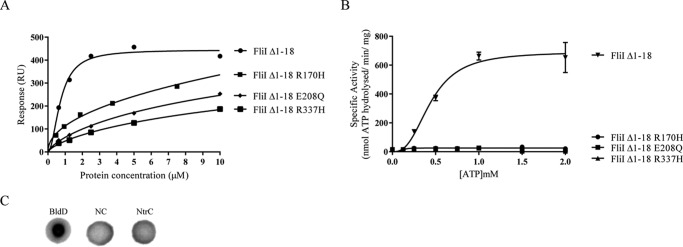
*A*, affinity fit for cdG binding to different FliI alleles (FliI_Δ1–18_, FliI_Δ1–18_ R170H, FliI_Δ1–18_ E208Q, and FliI_Δ1–18_ R337H). *B*, ATPase activity for different FliI alleles. Protein specific activity in each case (nmol ATP hydrolyzed/min/mg protein) is shown for increasing ATP concentrations. *C*, DRaCALA binding assay for [^32^P]cdG to 10 μm NtrC (*A. vinelandii*). Positive (10 μm BldD*) and negative binding controls (*NC*) were included as appropriate.

To confirm the importance of these residues to cdG binding, specific amino acid substitutions were produced (R170H, E208Q, and R337H) in FliI_Δ1–18_. The solubility of the resulting FliI_Δ1–18_ alleles was confirmed by gel filtration (data not shown), and then cdG binding and ATPase activity were tested. All three substitutions showed seriously compromised cdG binding, with *K_D_* values that were too high to be accurately determined (Fig. 8*A* and Table 3) and a complete abolition of ATPase activity (Fig. 8*B*). Finally, we detected no cdG binding for the hexameric ATPase protein NtrC from *Azotobacter vinelandii*, which shares a tertiary structure fold with the export ATPases but does not have the residues of the proposed binding site (Fig. 8*C*). Together, these results strongly indicate that the binding indeed occurs at the proposed site shown in Fig. 7*D*.

## Discussion

Here we show that the second messenger cdG binds to the bacterial flagellum export ATPase FliI. This cyclic dinucleotide binding is apparently widespread, with FliI homologs from multiple different bacterial species showing strong, concentration-dependent binding activity upon the addition of cdG. Excitingly, cdG binding at physiologically relevant (low micromolar) affinities was also determined for the closely related type III secretion exporter HrcN from *Pto* DC3000 and the much more distantly related *P. fluorescens* Type VI ATPase ClpB2. Our findings implicate cdG in the direct, allosteric regulation of both flagellar protein export and type III/type VI-mediated virulence for a range of pathogenic, commensal, and beneficial bacterial species.

**FIGURE 9.**
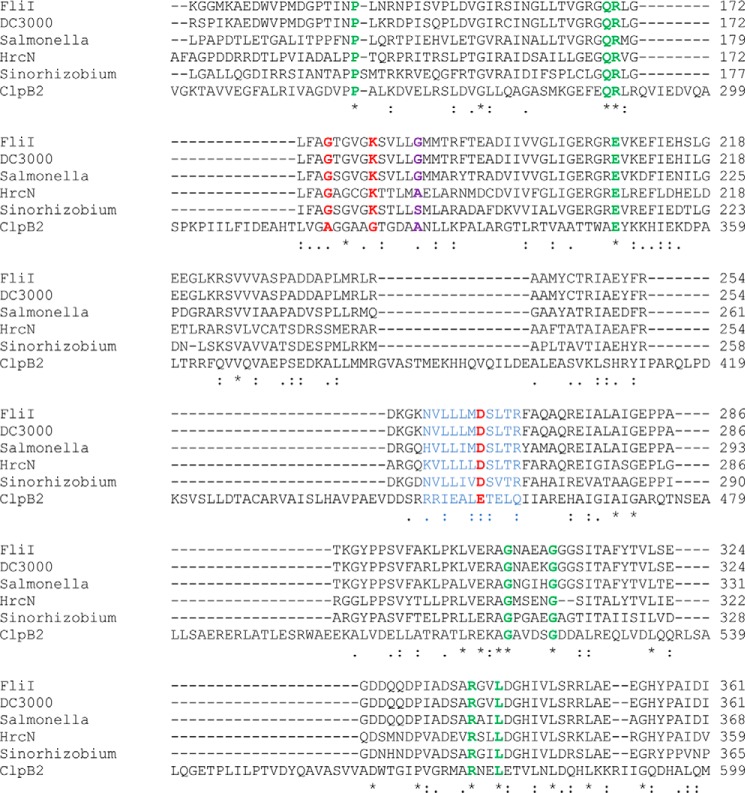
**Clustal alignment of conserved residues between FliI, HrcN, and ClpB2 proteins in this study.** Identities between all six residues are marked with *asterisks* (*), and similarities across all six with *periods* (.) or *colons* (:). The mutated FliI Walker A/B residues (see Fig. 6) are marked in *red*, the capture compound-binding NVLLLMDSLTR peptide is marked in *blue*, the position of the conserved cdG binding arginine in FlrA (Arg-176) is marked in *purple*, and the conserved residues of the proposed cdG binding site are marked in *green* (see Fig. 7).

The ATPase activity of both FliI and HrcN is suppressed by the addition of cdG. In this respect, the relationship between cdG and the export ATPase proteins is reminiscent of the transcriptional motility regulators FleQ (27) and FlrA (71). These proteins both contain AAA+ ATPase domains and bind cdG close to the Walker A motif of the protein. However, there appear to be important differences between the binding characteristics of FleQ/FlrA and the export ATPase proteins described here. In FleQ, cdG interacts with the Walker A site of the protein, leading to competitive inhibition of ATPase activity (27). Similarly, cdG binding to an arginine residue (Arg-176) downstream of the Walker A motif of FlrA inhibits binding to its target promoter sequence (71). In the case of the export ATPase proteins, we were able to uncouple ATPase activity from cdG binding. Our FliI/HrcN K181A mutants bound strongly to cdG but displayed no ATPase activity, whereas the G176A mutant retained substantial ATPase activity but showed severely compromised cdG binding. Furthermore, the FlrA R176 residue is conserved in FleQ but not in FliI/HrcN/ClpB2 (Fig. 9, *purple*), again suggesting a distinct cdG binding mechanism.

Based on our biochemical and mutagenesis data for FliI/HrcN, mass spectrometric analysis of cdG cross-linked FliI, and *in silico* modeling of hexameric FliI, we identified an intriguing potential site for cdG binding to the rotary export ATPases. This site, at the interface between two protein monomers, contains highly conserved glutamate and arginine residues known to be required for cdG binding (42). The putative binding pocket also contains both a proline and two glycines, which may play a role in maintaining the structure of the binding pocket. Mutagenesis and subsequent *in vitro* binding tests confirmed that the conserved glutamate and arginine residues were indeed required for cdG binding. Although loss of binding was accompanied by loss of ATPase activity in each case, this result is perhaps unsurprising given that the mutations made were at the FliI dimerization interface. Structural determination of FliI and HrcN in complex with cdG is underway and should allow us to determine exactly where and how cdG interacts with export ATPase complexes.

In general, cdG represses the production and function of flagella and type III secretion systems and promotes type VI secretion, although the relationship between cdG and these different pathways in *Pseudomonas* sp. is both highly complex and not fully understood. Flagella gene expression in *Pseudomonas* sp. is controlled by cdG through FleQ, which binds to numerous flagellar loci and whose inactivation by cdG binding (or deletion) abolishes flagella production (23). Flagella rotation is also likely to be under cdG control in *P. fluorescens* and *P. syringae*, because both contain close homologs to the *P. putida* rotation controller FlgZ (28). cdG has also been shown to control swarming motility in *P. aeruginosa* by switching between two different stator complexes; MotAB and MotCD (60). Other cdG-regulated pathways, such as pili synthesis (37) and exopolysaccharide production (23, 72) also have indirect impacts on both flagella deployment and motility (73, 74).

cdG is also known to control the level/activity of the *P. aeruginosa* type III and type VI secretion systems (64, 65), although here the regulatory pathways are less well understood. Moscoso *et al.* (65) show that cdG mediates the switch between production of type III and type VI secretion pathways and that this switch requires the sRNAs RsmY and RsmZ, linking cdG signaling to the small translational regulatory protein RsmA. Translation of both flagella and type III mRNAs are controlled by RsmA in *P. aeruginosa* (75), which is itself involved in a complex regulatory network involving downstream cdG signaling (76, 77). A role for cdG in the allosteric suppression of FliI/HrcN export *in vivo* is entirely consistent with the wider literature for both flagellar motility and type III secretion. Certainly, our biochemical data strongly suggest that increased intracellular cdG levels would suppress the ATPase activity and hence might be expected to suppress the export activity of these proteins. However, whether suppression of ATPase activity represents the actual *in vivo* function of export ATPase-cdG binding is currently uncertain.

Recently, Minamino *et al.* (14) showed that only residual FliI ATPase activity is actually required for flagellum production in *Salmonella*, with the majority of the energy for protein export provided by the proton motive force. FliI ATPase activity is still required for effective export to occur but is thought to play a gatekeeper role, where it provides the basal body with the energy required to initiate protein export (13, 14). If, as Minamino *et al.* suggest, reduced FliI ATPase activity upon cdG binding does not necessarily translate into reduced flagellar protein export, then what else might be the role of cdG? Our results show that cyclic dinucleotide binding is both widespread and highly conserved among export ATPases. Furthermore, the binding affinities we observed for cdG are sufficiently high that dinucleotide binding should occur frequently under “normal” environmental conditions.

We propose that FliI-cdG binding may play a more fundamental role in controlling flagella function and assembly. Specifically, a basal level of FliI-cdG binding may be required for the initiation of FliI export, via the promotion of multimerization, imposition of rotational asymmetry to the FliI hexamer (78) or another undefined mechanism. In support of this hypothesis, basal levels of cdG have been shown to be required for flagella synthesis in both *S. enterica* (79) and *Caulobacter crescentus* (80). In both cases, deletion of all GGDEF domain-containing proteins, and hence cdG, from the cell resulted in a loss of flagella-driven motility. In *Salmonella*, cdG removal led to increased expression of flagella basal body genes, but a severe defect in the export of FliC (79). Similarly for *C. crescentus*, the production of basal body proteins was unaffected by cdG removal, whereas class III and class IV gene expression was severely reduced (80). In this case, the reduced flagella gene expression could be explained by anti-σ factor-induced feedback upon the loss of flagellar export (81). Whether such a mechanism also applies to the ATPases of type III and type VI systems is unclear at this stage. Although the cdG-null strain of *S. enterica* showed a loss of virulence consistent with loss of T3SS function (79), more evidence is required to confidently propose a model for the relationship between cdG and HrcN/ClpB2 function. Research is ongoing to determine the exact nature of the relationship between cdG and the rotary ATPase proteins and the impact of cdG-ATPase binding on motility and virulence in bacterial species.

## Author Contributions

E. T. conceived and designed the study, conducted most of the experimental work, produced Figs. 1–6 and 8, analyzed data, and contributed to writing the manuscript. C. E. M. S. contributed to the SPR work and relevant data analysis. D. M. L. produced Fig. 7, including modeling, figure preparation, and data analysis. T. W. conducted the in silico MS-PSA analysis and produced supplemental Fig. S1. R. H. L. purified proteins and provided technical assistance and support to this study. J. G. M. conceived and designed the study, produced Fig. 9, and wrote the manuscript.

## Supplementary Material

Supplemental Data

## Footnotes

3 The abbreviations used are:

T3SS: type III secretion system
SPR: surface plasmon resonance
cdG: cyclic di-GMP
DRaCALA: differential radial capillary of ligand assay
MS-PSA: mass spectrometry peak-shift analysis.
