# Supplementary material for: Bacterial Rotary Export ATPases Are Allosterically Regulated by the Nucleotide Second Messenger Cyclic-di-GMP
Source: J Biol Chem. 2015 Aug 11;290(40):24470–83. doi: 10.1074/jbc.M115.661439 (PMC4591828; doi:10.1074/jbc.M115.661439)
Supplement: Supplemental Data [file supp_290_40_24470__index.html]

Bacterial Rotary Export ATPases are Allosterically Regulated by the Nucleotide Second Messenger Cyclic-di-GMP — Bacterial Rotary Export ATPases Are Allosterically Regulated by the Nucleotide Second Messenger Cyclic-di-GMP — Cyclic di-GMP Binding to Bacterial Export ATPases — Supplemental Data 

# Bacterial Rotary Export ATPases Are Allosterically Regulated by the Nucleotide Second Messenger Cyclic-di-GMP

## Supplemental Data

- Figure S1 legend (.pdf, 5 KB) - This is the legend for figure S1
- Figure S1 (.pdf, 25.9 MB) - This is a very large .pdf that shows the MS-PSA analysis. The .pdf cannot be displayed at manuscript page size without losing all readability.
