# Supplementary material for: Bacterial Rotary Export ATPases Are Allosterically Regulated by the Nucleotide Second Messenger Cyclic-di-GMP
Source: J Biol Chem. 2015 Aug 11;290(40):24470–83. doi: 10.1074/jbc.M115.661439 (PMC4591828; doi:10.1074/jbc.M115.661439)

SUPPLEMENTARY FIGURE 1A

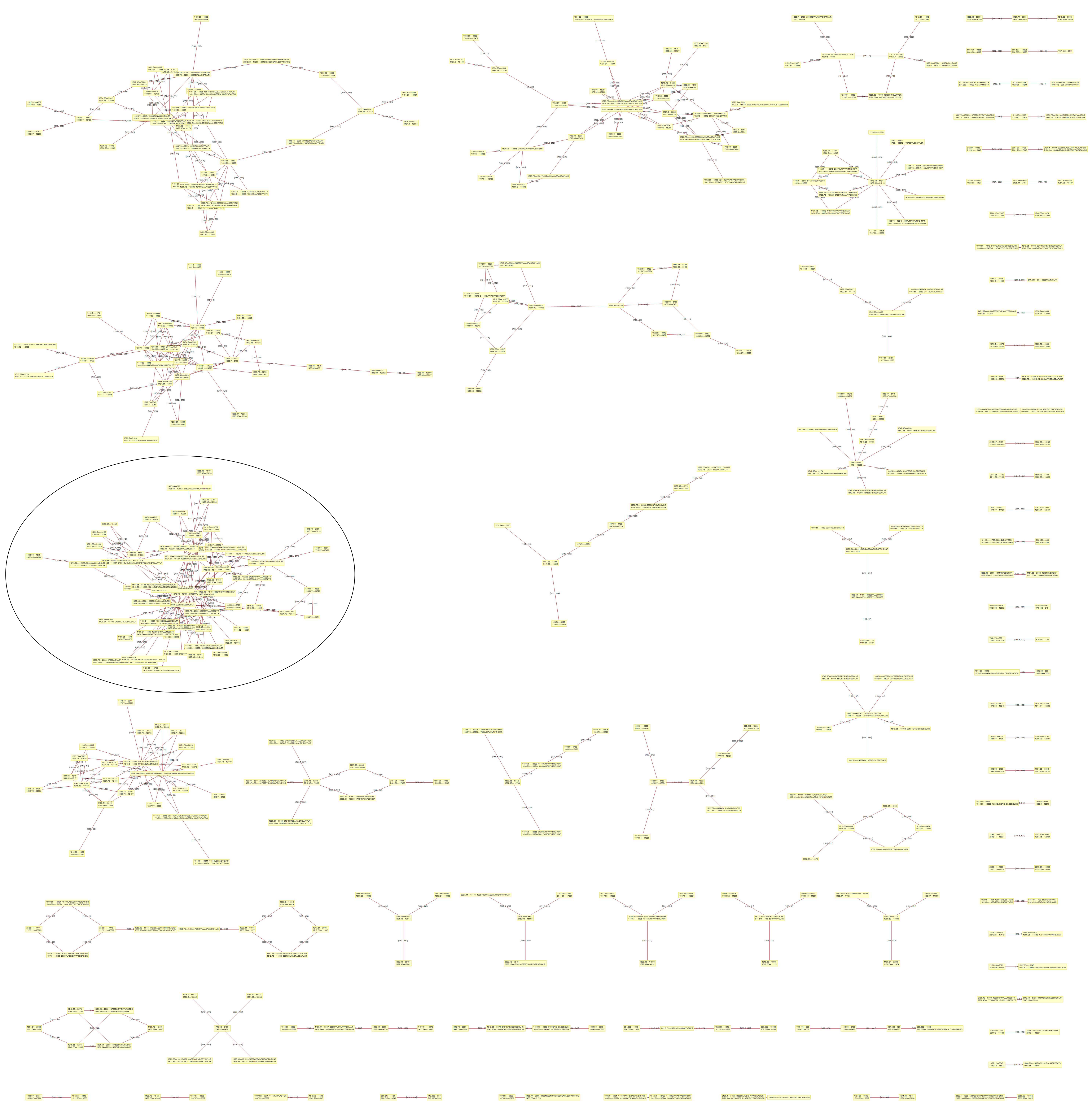

SUPPLEMENTARY FIGURE 1B

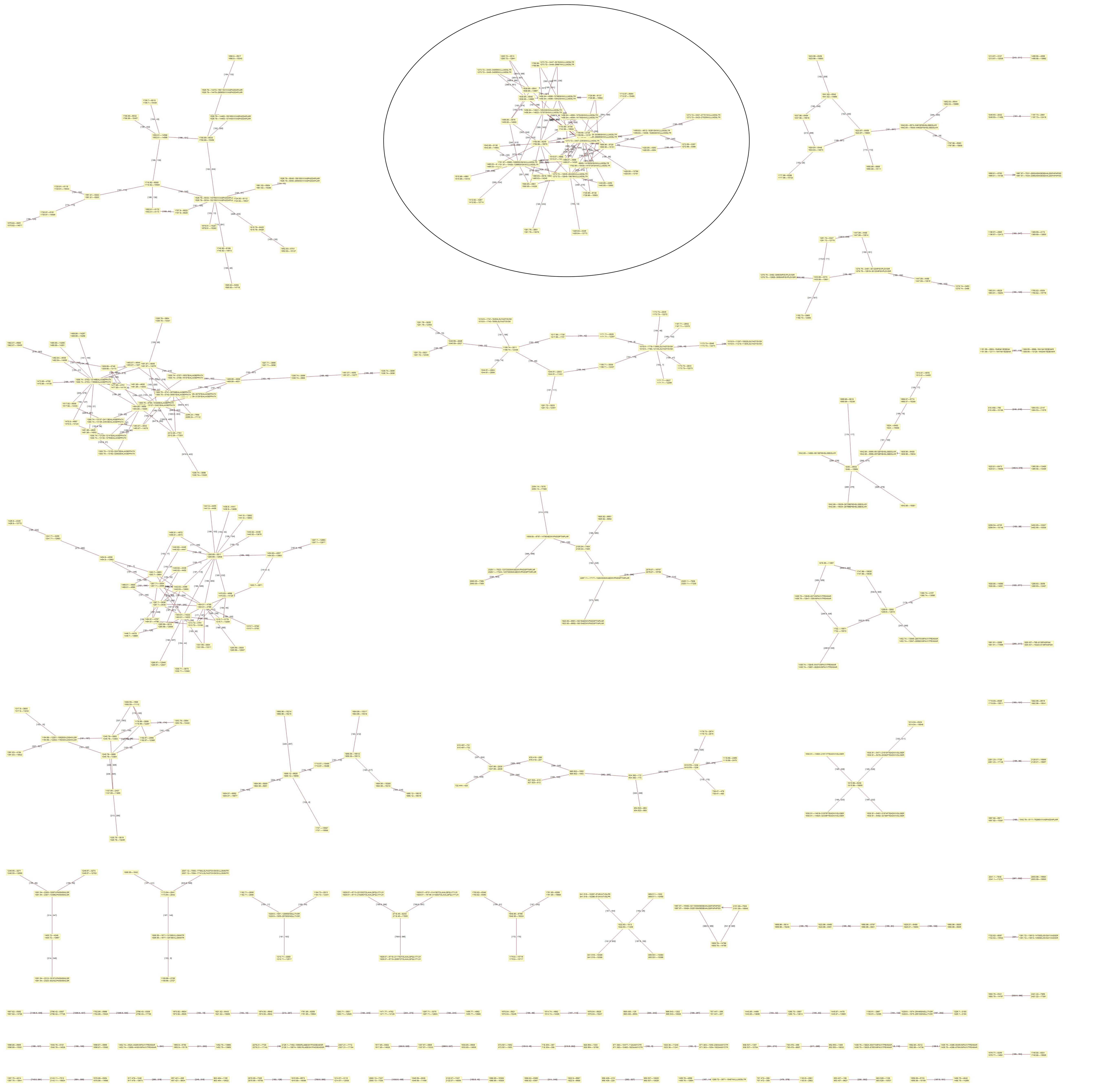

Supplement: Supplemental Data [file supp_M115.661439_jbc.M115.661439-2.pdf]
