# Supplementary material for: Bacterial Rotary Export ATPases Are Allosterically Regulated by the Nucleotide Second Messenger Cyclic-di-GMP
Source: J Biol Chem. 2015 Aug 11;290(40):24470–83. doi: 10.1074/jbc.M115.661439 (PMC4591828; doi:10.1074/jbc.M115.661439)
Supplement: Supplemental Data [file supp_M115.661439_jbc.M115.661439-1.pdf]

Bacterial Rotary Export ATPases are Allosterically Regulated by the Nucleotide Second Messenger Cyclic-di-GMP

Eleftheria Trampari[1], Clare E.M. Stevenson[2], Richard H. Little[1], Thomas Wilhelm[3], David M. Lawson[2] and Jacob G. Malone[1, 4, *]

SUPPLEMENTARY FIGURE

Figure S1 (attached .pdf file): Mass Spectrometry Peak Shift Analyses (MS-PSA) for treated (i.e. cross-linked to cdG) vs untreated $FliI_{His}$ (S1A) and treated $FliI_{His}$ only (S1B). The particularly dense clusters of related spectra in both analyses correspond to the most densely-modified peptide following cdG-capture compound crosslinking. In both cases, this peptide, (NVLLLMDSLTR) comprises residues 259-269 of FliI.
